# Social learning in otters

**DOI:** 10.1098/rsos.170489

**Published:** 2017-08-30

**Authors:** Zosia Ladds, William Hoppitt, Neeltje J. Boogert

**Affiliations:** 1 Department of Life Sciences, Anglia Ruskin University, Cambridge, UK; 2 School of Biology, University of Leeds, Leeds, UK; 3 Centre for Ecology and Conservation, University of Exeter, Penryn, UK

**Keywords:** group living, network-based diffusion analysis, otters, problem-solving, social learning, social networks

## Abstract

The use of information provided by others to tackle life's challenges is widespread, but should not be employed indiscriminately if it is to be adaptive. Evidence is accumulating that animals are indeed selective and adopt ‘social learning strategies’. However, studies have generally focused on fish, bird and primate species. Here we extend research on social learning strategies to a taxonomic group that has been neglected until now: otters (subfamily Lutrinae). We collected social association data on captive groups of two gregarious species: smooth-coated otters (*Lutrogale perspicillata*), known to hunt fish cooperatively in the wild, and Asian short-clawed otters (*Aonyx cinereus*), which feed individually on prey requiring extractive foraging behaviours. We then presented otter groups with a series of novel foraging tasks, and inferred social transmission of task solutions with network-based diffusion analysis. We show that smooth-coated otters can socially learn how to exploit novel food sources and may adopt a ‘copy when young’ strategy. We found no evidence for social learning in the Asian short-clawed otters. Otters are thus a promising model system for comparative research into social learning strategies, while conservation reintroduction programmes may benefit from facilitating the social transmission of survival skills in these vulnerable species.

## 1. Introduction

Animals can use personally acquired information (e.g. through trial-and-error learning) or information provided by others to guide their daily decisions. The latter, termed ‘social information’, can affect, for example, what, where or when animals eat, whom they mate with and how they respond to predators [1]. Research on social learning has come a long way since the phenomenon was first described by Aristotle in the fourth century BC [1]. Theory predicts that indiscriminate social information use is unlikely to be adaptive [2]. In recent years, various studies have focused on the possibility that animals may adopt ‘social learning strategies’ [3,4], such as when to copy the behaviour of others rather than learning asocially (‘when’ strategies), and whose behaviour to copy (‘who’ strategies).

‘Who’ strategies that involve copying kin may be adaptive [2,5]. The rationale for the ‘copy kin’ strategy is that social learning may be most useful when observers and demonstrators share the same environment, and individuals sharing local environments are often related. In addition, demonstrators may have more to gain from providing accurate information to kin than to non-kin [3]. However, empirical evidence for the ‘copy kin’ social learning strategy is mixed: while some species only copy the food choices from those genetically related and/or familiar to them (e.g. Mongolian gerbils [6]), others are more likely to copy the food choices of unfamiliar individuals (e.g. Norway rats [7,8]).

Copying older individuals is another ‘who’ strategy that one would expect to be favoured, as old age signals survival success. Evidence for this social learning strategy comes from guppies, for example, where small (and thus younger) females will copy the mate choices of older, larger, females, while the latter are not influenced by the mate choices of younger demonstrators [9]. More recent evidence comes from, for example, nine-spined sticklebacks [10], chimpanzees [11,12], meerkats [13], blue tits [14] and zebra finches [15]. Weanling Norway rats are also more likely to copy the food choices of adults rather than juveniles [16]. In other cases, however, young Norway rats were found to be equally likely to learn from both young and old demonstrators [17].

With regards to ‘when’ strategies, theory suggests that social learning should be used in novel situations when pre-existing or established behaviour is unproductive, and asocial learning may be costly [2,18,19]. For example, callitrichid monkeys used social learning strategies only to solve the most difficult novel extractive foraging tasks [20]. Similar patterns might be observed when individuals follow a ‘copy when uncertain’ strategy, where they copy others in unfamiliar or changing conditions where the best action to take, or the solution to the novel challenge encountered, is unknown to them. The ‘copy when uncertain’ strategy is predicted by theory [18], and there is evidence for its use in various animals (e.g. [21,22]). For similar reasons, we might expect younger individuals to be more reliant on social learning (i.e. a ‘copy when young’ strategy), because they have less experience of the world, and so are likely to be generally more ‘uncertain’ in an informational sense. Young meerkats, for example, were more likely than adults to copy the location where conspecifics were interacting with a novel foraging apparatus [23], and younger female guppies copied the mate choices of older females, but not vice versa [9].

Previous empirical studies thus suggest that the particular ‘who’ and ‘when’ social learning strategies used depend on various factors. These determinants and resulting social learning strategies may differ between species, depending on, for example, opportunities for social interaction, the extent to which behaviour needs to be adjusted to changing spatial and/or temporal environments, and how risky or costly it is to obtain personal information [24]. However, the great majority of such research has focused on a limited number of primate [25], fish [26], bird [24] and insect species [27]. Here we extend research on social learning strategies to a taxonomic group that has been entirely neglected until now, but in which various social learning strategies are expected to be manifested: otters.

Otters show a great diversity of social systems among the 13 species, with some being fiercely territorial and others living in large family groups [28]. Otters also exhibit a wide range of foraging behaviours as they handle many different food types. The diet of smooth-coated otters, for example, consists almost entirely of fish, which they catch using coordinated hunting strategies [28]. Asian short-clawed otters, on the other hand, eat mainly crabs, and use their long, clawless fingers to feel around in silt and crevices for shellfish and other invertebrates. The otters then crush the shells using their teeth [28]. Given the variety of dietary and foraging specializations observed in different otter populations [29], it seems plausible that they may be socially transmitted between individuals [30].

Smooth-coated otters and Asian short-clawed otters also differ in their social group structure and their reliance on social foraging in nature: wild smooth-coated otters typically live in social groups of four to five individuals, consisting of an adult female and her offspring from several litters, where the older offspring help to raise new litters. These family groups hunt cooperatively and show group defence against predators [28]. By contrast, Asian short-clawed otters live in large social groups of up to 15 individuals, consisting of one female and her offspring [28]. They have been studied less in the wild than the smooth-coated otters, and their social behaviours are not particularly well documented. It is thought that they generally forage individually for crabs and shellfish, but coordinate predator defence within their groups [28].

Surprisingly, it is not known whether these, or any other species of otter, can learn socially. Owing to their vulnerable conservation status [31,32], a better understanding of otter social learning tendencies could have valuable implications for future reintroduction programmes, as has been seen in reintroduction cases of, for example, hatchery-reared fish [33] and prairie dogs [34]. Training of anti-predator behaviour and foraging skills, for example, may be more efficient and effective when involving conspecific demonstrators [33,34]. Furthermore, research into otters' social learning strategies in terms of whom and when they copy could inform which type of demonstrator (e.g. young/old, male/female) to use and under which circumstances (e.g. when unfamiliar prey requires complex manipulation to exploit).

We presented novel extractive foraging tasks to captive groups of smooth-coated otters (*Lutrogale perspicillata*) and Asian short-clawed otters (*Aonyx cinereus*). We used network-based diffusion analysis (NBDA) [35,36] to infer that individuals socially learned the task solutions from each other if the diffusion (spread) of the task solutions through the otter groups followed the groups’ previously determined social association networks. Given that both smooth-coated and Asian short-clawed otters live in stable family groups and are thought to rely on each other for foraging and/or anti-predator defence, we predicted that both species would be capable of socially learning the solution to novel extractive foraging tasks. We also assessed whether the otters used (a) the ‘copy when asocial learning is costly’ strategy, by testing whether individuals' reliance on social learning increased with the difficulty of the novel foraging task presented, and (b) the ‘copy when young’ (and thus uncertain) strategy, by testing whether offspring were more likely to learn socially than their parents.

## 2. Material and methods

### 2.1. Study populations

We studied one group of smooth-coated otters (*N* = 7 individuals) and three groups of Asian short-clawed otters (*N* = 5, 6, and 6 individuals) at zoos and wildlife parks in the United Kingdom (see electronic supplementary material, table S1, for otter group compositions).

### 2.2. Association data

To calculate the social network for each group, we collected five consecutive days of association data. We distinguished individuals using differences in body size and shape and facial marks. We used spatial proximity as an indicator of social association [37]: if otters were within one body length of each other (a metric suggested in [37]), they were classified as associating. We collected the data for three separate periods of one hour on each of the five days, during which we recorded every 5 min which otters were associating. We used these data to create an association network for each otter group. Each otter dyad's ‘association index’ was computed as the simple ratio of the total number of 5-min samples that the two otters were observed within one body length of each other, divided by the total number of 5-min samples those individuals were observed apart plus the samples in which they were associating (the ‘simple ratio index’ [38]). We use the coefficient of variation of the associations in each network to provide a measure of social differentiation [39]. Although the 5-min social association samples in the same observation hour were not independent of each other, the number of sampling periods to quantify the social association network does not enter into the NBDA that tests for social learning (see Data analysis section). There is, therefore, no issue of observation period ‘pseudo-replication’ somehow biasing the network-based results. Furthermore, any noise in the social network data reduces the power to detect social learning using NBDA and thus results in a more conservative estimate of the importance of social learning rather than a false positive [40].

### 2.3. Diffusion experiments

**Figure 1.**
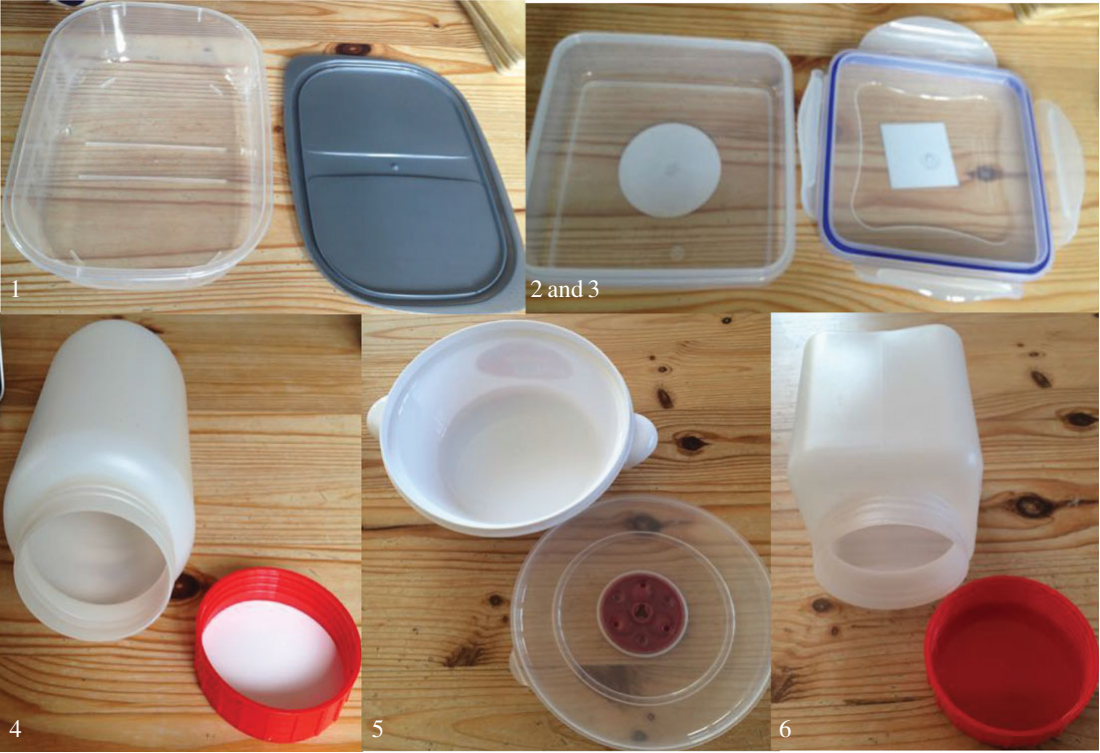
The novel extractive foraging tasks presented to the smooth-coated otters. (1) A simple plastic box (55 × 175 × 130 mm); (2) a plastic box with clips on the lid (45 × 135 × 135 mm); this box was used as both tasks 2 and 3, once with four clips closed (task 2) and then with two clips closed and two clips removed (task 3); (4) a round plastic jar with screw-top lid (180 × 90 mm diameter); (5) a round plastic tub with a pull-off lid (70 × 170 mm diameter); and (6) a square plastic jar with screw-top lid (180 × 85 × 85 mm). Each task was baited with half a fish and covered with its respective lid. The tasks are numbered in assumed order of difficulty with 1 being the easiest and 6 being the hardest.

**Figure 2.**
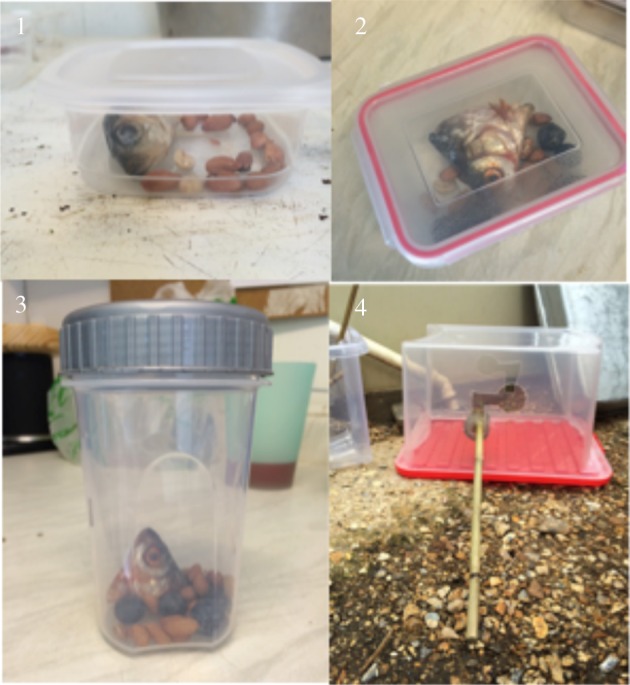
The novel extractive foraging tasks presented to the Asian short-clawed otters. These differ from those presented to the smooth-coated otters (figure 1), as in this second iteration of the experiment on the second study species we wanted to create a more explicit range of task difficulties. Tasks were also slightly smaller to accommodate the smaller size of this otter species. (1) A simple plastic box (40 × 100 × 100 mm); (2) a plastic box with two clips (45 × 100 × 85 mm); (3) a plastic tub with a screw-top lid (130 × 75 mm diameter); and (4) a frozen reward on a bamboo cane that had to be moved up and to the right to fit through the hole. The numbering of the tasks is in intended order of difficulty, with 1 being the easiest, and 4 being the most difficult. These tasks were baited with peanuts and one fish head per task at the New Forest Wildlife Park, and peanuts and mealworms mixed with either half a mouse or day-old chick legs at Paradise Wildlife Park. Task 4 required a different type of reward due to its design, so this was a block of ice with shrimp or mealworms frozen inside.

Next we introduced new information to each otter group, in the form of novel extractive foraging tasks baited with desirable food rewards (e.g. pieces of fish; see figures 1 and 2). Several identical replicas of each task type (usually two or three task apparatuses more than the number of otters in each group) were placed into the enclosure at the start of each diffusion experiment, so that each otter could have a chance to solve the task, and the otters could easily observe each other solving the tasks (see supplementary videos 1--3 at the Dryad Digital Repository [41]). Each task type was presented on a separate occasion to each group of otters, resulting in six ‘diffusions’ of the solutions to six different task types in the group of seven smooth-coated otters (figure 1), and four ‘diffusions’ of the solutions to four different task types in each of the three groups of Asian short-clawed otters (figure 2). We predicted that the otters would have more trouble solving the task types that, from our personal experience opening the tasks, required more complex extractive foraging techniques (e.g. figure 1 tasks 5–6; figure 2 tasks 3–4), and would thus be more inclined to copy each others' solutions to those task types.

One task type was presented per day in between the otters’ regular feedings by the wildlife centres, approximately 2 h after the normal food was last provided. The task apparatuses were left inside the enclosure until either they had all been solved (i.e. the food reward removed), or none of the otters had interacted with the task apparatuses for 3 h. If the latter occurred, the same task type was presented again the following day. A ‘solve’ was recorded every time an otter managed to retrieve the food reward from a task apparatus. If another member of the group stole the reward, the otter that originally opened the task apparatus was recorded as solving the task. The order in which the otters solved each task type was recorded. It was not possible for the single experimenter (Z.L.) observing the otters from outside the enclosure to accurately record the exact time at which each individual solved each task, as otters would pick up the task apparatuses and take them to different parts of the enclosure. However, we were able to record in discrete time the minute in which each otter solved each task type. The raw data have been uploaded as part of the electronic supplementary material.

### 2.4. Data analysis

NBDA infers that individuals acquire novel behaviours socially if the order (order of acquisition diffusion analysis: OADA) or time sequence (time of acquisition diffusion analysis: TADA) in which the individuals start showing the novel behaviour follows their social association network [36]. In other words: if individual A associates often with individual B but rarely with individual C, we would infer social transmission if individual A started performing a novel behaviour following B's, but not C's, demonstration of that behaviour.

We initially used the OADA variant of NBDA to determine whether the order in which subjects solved the tasks was correlated with the pattern of their social associations. OADA makes fewer assumptions than the TADA variant of NBDA [36], and here we did not have exact times of acquisition, so we initially used OADA. However, the social networks for Asian short-clawed otters were highly homogeneous (see Results), meaning OADA was unable to estimate the effect of social transmission with precision. We, therefore, also used a discrete TADA [35,36] to estimate the effects of social transmission for this species, with time divided into 1-min periods (see electronic supplementary material). This also allowed us to estimate the relative difficulty of each task for the Asian short-clawed otters.

In both OADA and (discrete) TADA, inference of social transmission results from comparing models that include social transmission to models with only asocial learning. Each model also contained age category (i.e. parent (coded as ‘1’)/offspring (coded as ‘0’)) and sex (female coded as ‘1’, male as ‘0’) as predictor variables. To assess whether otters differed in their reliance on social learning among tasks (e.g. they may have increased reliance on social learning with increasing task difficulty), we compared model fits assuming social transmission rates were the same versus different for all tasks. We also fitted both additive and multiplicative versions of each social transmission model [36]. The additive model assumes that differences between ages or sexes apply only to asocial learning, whereas the multiplicative model assumes that differences apply to both social transmission and asocial learning.

We used a model-averaging approach with Akaike's information criterion, corrected for small sample size, to select the best model [42]. For each variable considered, we give its total Akaike weight (as a percentage) and model-averaged estimate. We also provide unconditional 95% confidence intervals using Burnham and Anderson's method for adjusting profile likelihood confidence intervals for model selection uncertainty [42]. We use Akaike weights to quantify the relative support for the different models of social transmission and the asocial learning model. To give a more intuitive measure of the strength of social transmission, we derive estimates of the proportion of task solutions that occurred by social transmission [43].

## 3. Results

### 3.1. Smooth-coated otters

**Figure 3.**
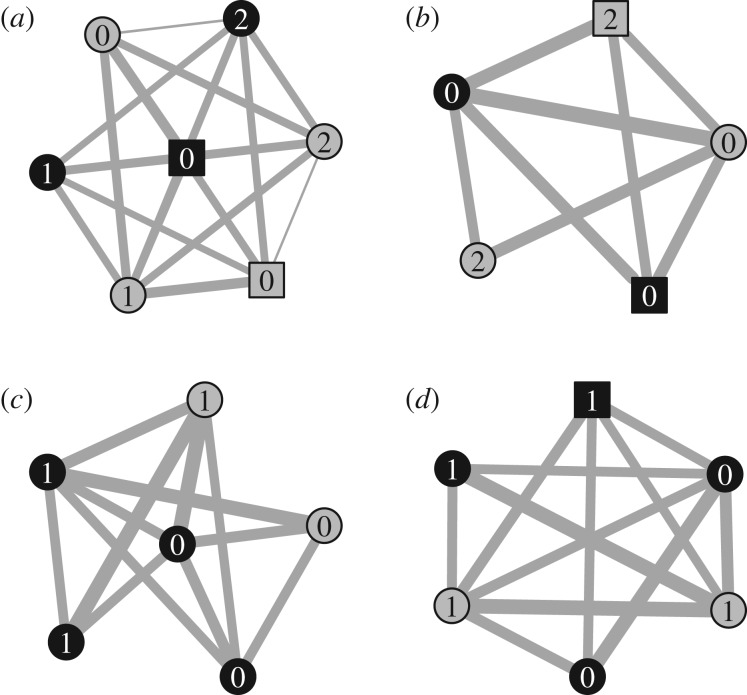
Association networks for the four otter groups. (*a*) Smooth-coated otters; (*b*–*d*) Asian short-clawed otters. The line thicknesses are scaled according to the strength of association between each dyad of otters. Black nodes represent males, grey nodes represent females; square nodes represent parents, circular nodes represent offspring. The numbers on the nodes represent the number of times each otter was the first individual to solve a task (i.e. the innovator).

The network structure (figure 3*a*) shows that although virtually all members of the group associated with each other (i.e. almost all network nodes are connected), individuals differed greatly in how much they associated with each other (i.e. different line thicknesses between nodes in figure 3*a*; social differentiation coefficient = 0.53).

Most smooth-coated otters solved all task types (electronic supplementary material, table S2; and supplementary video 1 [41]). The otters in this group appear to have solved the tasks by copying each other rather than through individual trial-and-error learning: there was 6.5× more support for the best of the social transmission models (Akaike weight: 79.4%) than for the asocial learning model (Akaike weight: 12.3%; table 1), indicating that the solutions to the novel foraging tasks spread through the otter group following the association network. The rate of social transmission per unit network connection, relative to the baseline rate of asocial learning, was estimated to be 45.2 (95% CI = [0.44, ∞]). This corresponds to the otters (except for the first solver or ‘innovator’ in the group) using social information in 96.0% (95% CI = [27.5, 100]) of all task solves (table 2).

**Table 1.** A comparison of the support (based on Akaike weight) for different social learning models and the asocial learning model. Italicized variables indicate the models with most support.

model form	rate of social transmission same/different across tasks	smooth-coated otters	Asian short-clawed otters
asocial		12.3%	*59*.*4%*
social transmission			
additive model	same	7.0%	20.1%
	different	0.8%	1.5%
multiplicative model	same	*79*.*4%*	19.6%
	different	0.6%	0.5%

**Table 2.** Estimates of the effect of social transmission in each species.

species	social transmission rate per unit network connection(s)	per cent of events by social transmission (excluding innovation)
smooth-coated otters	45.2 (0.44–∞)^a^	96.0% (27.5–100%)
Asian short-clawed otters	dTADA: 0 (0–0.17)^b^	dTADA: 0% (0–17.3%)

^a^Note that it is common for OADA to fail to put an upper limit on the strength of social transmission when the order of learning follows the network closely. This simply means it is plausible that all individuals learned by social transmission, except the innovator.

^b^OADA was unable to precisely estimate the effect of social transmission in Asian short-clawed otters, so we also ran a dTADA for this species (see main text and electronic supplementary material, table S4).

**Figure 4.**
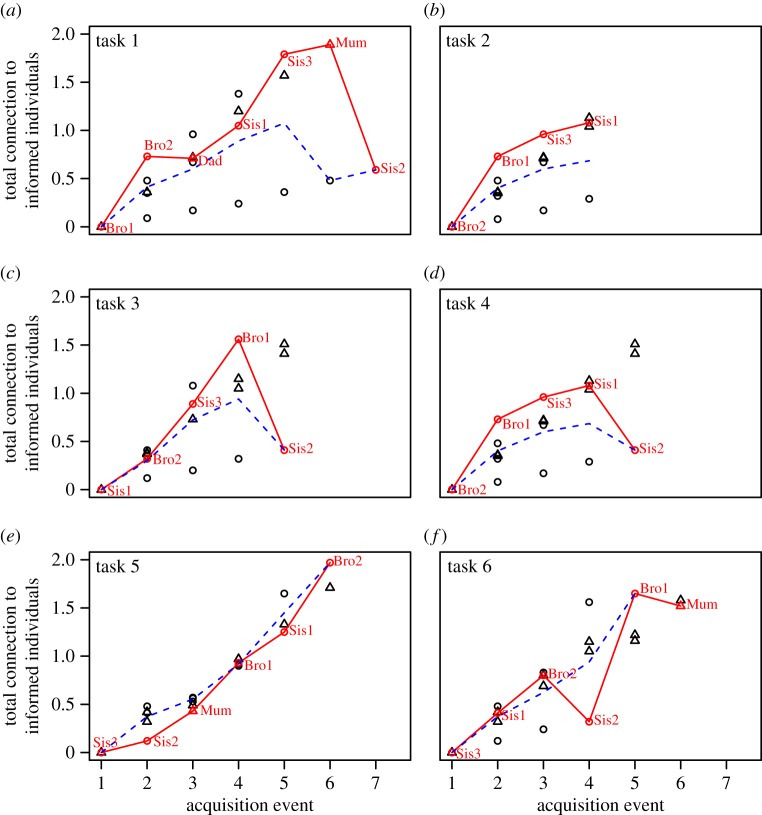
The order of solving in the smooth-coated otters for each of the six tasks. The total association of each naïve individual with informed individuals is plotted against the sequence of solving (task-solving event = 1 is when the first individual solved, etc.). Offspring are plotted as circles and parents as triangles. The individual that solved the task at each part of the sequence is plotted in red and joined with red lines. The dashed blue line shows the path we would expect the red line to take if there were no social transmission (but allowing that juveniles are faster to solve). If there is social transmission through the network the red line should be above the blue line. The solvers are labelled as Mum/Dad for parents and as Sis (sister)/Bro (brother) with ID number for offspring. In (*a*–*d*) tasks 1–4, the individual to solve the task next tends to be an offspring with a relatively high level of association to informed otters relative to others for that solve, and the red line is clearly above the dashed blue line. In (*e*–*f*) tasks 5–6, this pattern is no longer apparent.

The smooth-coated otters do not seem to have adopted a ‘copy when asocial learning is costly’ strategy: the solving data were best described by models that specified an equal social transmission rate across tasks (table 1), instead of a varying rate that might have indicated increased reliance on social transmission for more complex tasks. However, an ad hoc visual examination revealed that the spread of task solutions through the otter group seemed to follow the association network more closely in task types 1–4 than 5–6 (figure 4; supplementary video 1 [41] shows a task 5 trial). When inspecting the sequence in which otters solved task types 1–4, the next individual to learn to solve a task tended to be a young otter that was one of the most strongly associated in the social network with ‘informed’ group mates, i.e. those that had already solved the task. In task types 5–6 this pattern was no longer apparent (figure 4). Furthermore, fitting a model *without* social transmission to the solving-order data for tasks 5 and 6 improved Akaike's information criterion (corrected for small sample size) by 4.41, suggesting that smooth-coated otters may have learned individually rather than socially how to solve task types 5 and 6.

The social transmission model with most support was multiplicative rather than additive (11.3× more support; table 1). This means that any sex/age differences in solving the tasks affected both asocial and social learning rates, rather than just asocial learning. There was little support for smooth-coated otter males differing from females in task-solving speed (total Akaike weight = 24.9%; table 3), with females being an estimated 1.02× (95% CI = [0.63, 1.65]) faster to solve tasks than males. However, there was strong support for a difference between offspring and parents (total Akaike weight = 100.0%; table 3), with offspring being an estimated 6.10× (95% CI = [1.11, 33.53]) faster to solve the tasks than the parents. The parents only solved 1 out of 6 tasks (father) and 3 out of 6 tasks (mother), compared with the offspring solving an average of 5.8 tasks. Given the stronger support for the multiplicative model (table 1), this indicates that young otters learned faster how to solve tasks both socially and asocially when compared with their parents.

**Table 3.** Support (total Akaike weight) and estimates for the effects of sex and age. Italicized variables indicate evidence for a sex/age effect on social transmission.

	smooth-coated otters		Asian short-clawed otters		
effect	support	MAE (95% CI)^a^	support	MAE (95% CI)^a^	ratio
sex (female/male)	24.9%	1.02 × (0.63–1.65)	*92*.*8%*	*2.26 × (1.12–4.54)*	ASC/SC = 2.22 × (0.71–6.95)
offspring/parents	*100*.*0%*	*6.10 × (1.11–33.53)*	27.1%	0.95 × (0.56–1.62)	*SC/ASC = 7.48* × (*1.38–40.64*)

^a^MAE, model-averaged estimate averaged across the best supported set of models for that species (i.e. the cell with highest support in table 1), with back-transformed Wald 95% confidence intervals based on unconditional standard errors. The ratios of effects are taken from a combined OADA model for both species, including social transmission, sex and age, all of which were allowed to differ between species.

### 3.2. Asian short-clawed otters

The network diagrams are much more homogeneous for the three groups of Asian short-clawed otters than for the smooth-coated otters (figure 3*b,c,d* versus *a*; social differentiation coefficients = 0.16, 0.17, 0.32 versus 0.53) suggesting that Asian short-clawed otters did not differ as much as the smooth-coated otters in how frequently individuals associated with each other. Fewer than half of the otters solved task 4 (figure 2; electronic supplementary material, table S3; supplementary video 3 [41]), suggesting that this was indeed the most complex task. NBDA (TADA) confirmed that tasks 3 and 4 were more difficult (slower) to solve than tasks 1 and 2 (expected time to solve relative to task 1: task 2= 2.45× faster; task 3= 0.33× slower; task 4= 0.42× slower).

The individuals in the three Asian short-clawed otter groups appear to have learned the solutions to the four novel foraging task types individually rather than socially: there was almost 3× more support for the asocial learning model (Akaike weight: 59.4%) than for the best-fitting social transmission model (Akaike weight: 20.1%; table 1). The rate of social transmission per unit network connection, relative to the baseline rate of asocial learning, was estimated at 0 (95% CI = [0, 0.17]), corresponding to 0% (95% CI = [0, 17.3%]) of task solutions occurring due to otters (excluding the innovator) socially learning from each other (table 2). There was also no support for a ‘copy when asocial learning is costly’ strategy, as the task-solving data were best described by models that specified a social transmission rate that was equal across all tasks (table 1). Together, these results suggest that Asian short-clawed otters are unlikely to have relied on social information to solve the novel foraging tasks.

In contrast to the smooth-coated otters, Asian short-clawed otter parents and offspring did not appear to differ in their task-solving speeds (total Akaike weight = 27.1%), with offspring being an estimated 0.95× (95% CI = [0.56, 1.62]) slower than parents (table 3). However, there was strong support for a difference between sexes (total Akaike weight = 92.8%), with females being an estimated 2.26× (95% CI = [1.12, 4.54]) faster to solve the tasks than males (table 3).

### 3.3. Species comparison

There was strong evidence for social transmission in smooth-coated otters, but not in Asian short-clawed otters (tables 1 and 2). Across all tasks presented to each species, Asian short-clawed otters were estimated to use social information in a maximum of 17.3% of all task solves, compared with a minimum estimate of 27.5% in smooth-coated otters (table 2), suggesting that smooth-coated otters were more reliant on social transmission than Asian short-clawed otters. However, because some of the tasks presented to each species were different, we re-ran analyses using only the tasks presented to both. When including only the three tasks that required the same actions to be solved (smooth-coated otters: tasks 1, 3, 4 (figure 1) and Asian short-clawed otters: tasks 1, 2, 3 (figure 2)), the NBDA estimated that smooth-coated otters (except for the innovators) used social transmission in 100% (95% CI = [45.8, 100]) of task solves compared to 0% (95% CI = [0, 20.7]) for Asian short-clawed otters. When the screw-top task was also excluded from the analysis (because a clear container was used for the Asian short-clawed otters (figure 2: task 3) and an opaque container for the smooth-coated otters (figure 1: task 4)), smooth-coated otters were estimated to use social transmission in 100% (95% CI = [15.3, 100]) of task solves compared with 20.4% (95% CI = [0, 59.5]) for Asian short-clawed otters. In all cases, the estimated effect of social transmission was thus much stronger for smooth-coated otters. However, in the latter most stringent comparison of the two species' performance on only two of the tasks, the 95% confidence intervals overlap. Therefore, overall our results strongly suggest that social transmission was less likely to be an important factor in the spread of task solutions in Asian short-clawed otters than in smooth-coated otters, but replication in a larger number of otter groups of both species presented with a larger battery of identical tasks is needed to confirm this finding.

Although males and females differed in task-solving rates only in Asian short-clawed otters, there is only weak support for a meaningful species difference: the sex difference in solving rates was estimated to be 2.22× stronger in Asian short-clawed otters when compared with smooth-coated otters, but with a 95% confidence interval including 1: [0.71, 6.95] (table 3). This indicates that we cannot exclude the possibility that the two species showed the same sex difference in solving rates. However, there was reasonable evidence that the relative age difference in task-solving rates, with young otters solving significantly faster than their parents, was stronger in smooth-coated otters than in Asian short-clawed otters (7.48×; 95% CI = [1.38–40.64]; table 3). A caveat here is that the offspring in the smooth-coated otter group were 1–2 years old, while the offspring in the Asian short-clawed otter groups were 4–10 years old. Whether younger Asian short-clawed otter offspring would have outperformed their parents as observed in the smooth-coated otters thus remains to be investigated.

## 4. Discussion

Social learning has been studied in many species, but never in otters, even though many otter species are likely to be capable of social learning given their gregarious nature, and knowledge of their social learning strategies may help inform reintroduction programmes to support these vulnerable species. The aim of our study was threefold: to address whether otters would (a) socially learn the solutions to novel extractive foraging tasks, (b) exhibit a ‘copy when asocial learning is costly’ strategy, and (c) show evidence of a ‘copy when young’ social learning strategy. We tested two species of otter that live in family groups but differ in life-history traits as well as diet. Given their gregarious nature, we predicted that both species may show evidence of social learning. However, we made no predictions concerning species differences or the adoption of particular social learning strategies, given the exploratory nature of this study and the fact that no one has studied social learning in otters before.

We show for the first time that smooth-coated otters can learn from each other how to solve novel foraging tasks, while we found no support for this in the Asian short-clawed otters. These results are based on only a few captive groups. As so little is known about these species in the wild, it is unclear how differences between captive groups and those in nature in factors such as demographic composition and individuals' experiences may affect the animals’ behaviour and reliance on social learning. Our findings regarding otter species and age differences in social learning tendencies should thus be interpreted with caution and would benefit from replication on a larger captive sample and validation in wild populations. Nonetheless, our results offer a first insight into the social learning abilities of the subfamily Lutrinae. Furthermore, our results make ecological sense if we consider what *is* known about the natural foraging habits of these species: smooth-coated otters show coordinated group-hunting strategies such as creek-wide aligned swimming patterns to catch fish [28,44]. However, their natural prey does not necessitate extensive extractive foraging behaviour to consume. In our experimental setting, it thus makes sense that the smooth-coated otters would be naturally inclined to watch each other for foraging information, especially as they are unlikely to have adapted to deal with complex extractive foraging tasks. In contrast, the Asian short-clawed otters are not known to forage in groups, and their natural diet consists mainly of prey (i.e. shellfish, crabs) that require extractive foraging techniques, but not group-hunting strategies, to consume [28]. They may, therefore, have less of a natural tendency to turn to each other when facing novel food puzzles that are somewhat similar to the prey they encounter in the wild. However, virtually no information is available on the development of extractive foraging behaviours in Asian short-clawed otters and the extent to which these are (socially) learned in the wild. Field studies have provided extensive evidence for juveniles' reliance on social learning to acquire extractive foraging behaviours in other mammal species, including black rats [45], meerkats [13] and chimpanzees [12]. We cannot exclude the possibility that Asian short-clawed offspring younger (≤2 years old) than those that were available to us (4–10 years old) might have relied on social information to solve our food puzzles. We have just acquired access to breeding populations of Asian short-clawed otters and additional populations of smooth-coated otters and hope to determine in the near future (a) to what extent newborn pups use social information across development to acquire their extractive foraging skills and (b) whether we can replicate or reject our preliminary finding of a species difference in reliance on social learning between smooth-coated and Asian short-clawed otters.

With regards to our second research question, we did not find any evidence that either otter species adopted the ‘copy when asocial learning is costly’ strategy, which we would have inferred had we found increased reliance on social learning with increasing task difficulty. In the smooth-coated otters, the order in which we presented the tasks was confounded with task difficulty (because we did not initially intend to address this question at the time of experimental design), such that the last two tasks to be presented also appeared to be the most difficult ones. *Post hoc* analyses actually showed that the order in which the otters solved the tasks followed the social network more closely for the first four tasks than for the latter two, and for the latter two a model including only asocial learning (i.e. without social transmission) provided a better fit to the data. On the face of it, this is contrary to the predictions of a ‘copy when asocial learning is costly’ strategy. However, the result may instead be due to the otters having gained sufficient experience with the previous four tasks that by the end of the experimental period they no longer relied on each other to solve them. To address these concerns, we counterbalanced the task order across the groups of Asian short-clawed otters, but again found no support for social transmission in this species, even for the most difficult task that fewer than half of the otters managed to solve. Based on these results we, therefore, draw the tentative conclusion that these two otter species may not adopt a ‘copy when asocial learning is costly’ strategy, although replication in several additional groups of both smooth-coated and Asian short-clawed otters with a randomized task presentation order is necessary to corroborate this conclusion. Even then, our results may be consistent with the strategic use of social learning in smooth-coated otters. Theoretical analyses [46] suggest that a ‘critical social learner’ strategy is often adaptive, whereby individuals try to copy others first and rely on trial-and-error learning only in situations where copying fails to yield satisfactory results (but see [47,48]). Further work might aim to investigate whether this strategy operates in otters.

Finally, our results suggest the possibility that smooth-coated otters adopt a ‘copy when young’ strategy: virtually all offspring solved all six tasks and did so over six times faster than their parents who solved half (mother) or only one (father) of the tasks. We found no such patterns in the Asian short-clawed otters. Again, further work is needed to be certain that this is a general difference between the species across a larger number of groups, as we cannot exclude the possibility that the adults and young in the single smooth-coated otter group we studied happened to differ in unrelated factors (e.g. motivation, fear of novelty, reinforcement to explore/play with novel objects in the environment) that could have generated an apparent age difference in reliance on social learning. Nonetheless, this apparent species difference in social learning strategies makes sense if we consider species differences in life-history traits: smooth-coated otters take almost double the time to reach sexual maturity and reproduce (when approx. 4 years old) when compared with the Asian short-clawed ones (when approx. 2 years old [28]). This extended juvenile period in the family group in smooth-coated otters is likely correlated with an extended period for (socially) acquiring essential skills for survival. Asian short-clawed otters on the other hand reach sexual maturity, and presumably independence from the family group, much faster, which may explain their apparent overall lower tendency to learn socially, as well as a lack of evidence for the ‘copy when young’ strategy. However, as noted above, it is important to consider again here that the youngest Asian short-clawed otter tested was already four years old, and the oldest offspring was ten. A valid criticism of our interpretation is that none of our test subjects in this species qualified as ‘young’ in the first place. We can thus conclude that, while smooth-coated otters do seem to adopt a ‘copy when young’ strategy, further studies on younger Asian short-clawed otters and additional smooth-coated otter groups are necessary if we are to make a fair species comparison. Furthermore, Asian short-clawed otters are known to have group-coordinated anti-predator behaviour [28]. It may well be that this species would show evidence for social transmission in tasks that tested the transmission of anti-predator behaviour against a novel stimulus [49–51] rather than foraging behaviour.

Our findings that smooth-coated otters are capable of learning from each other how to exploit novel food sources, and that there may be species differences in otters' reliance on social transmission, may have important conservation implications. Many otter species are listed as near threatened, vulnerable or endangered by the International Union for Conservation of Nature (IUCN). Conservation organizations facilitating reintroduction programmes could benefit from using social transmission as a way of training captive-bred otters to cope with life in the wild. Previous research suggests that animals trained on important life skills (e.g. anti-predator behaviour) through social learning have a higher survival rate once reintroduced into the wild [34]. Additional information on the captive animals’ social networks and social learning strategies may facilitate these efforts by pointing to the best individuals to seed with the information for it to be transmitted. For example, young smooth-coated otters' training before release into the wild may benefit from sibling demonstrators performing survival skills, as we found that the novel foraging task solutions spread most efficiently between peers (horizontal transmission) rather than from parents to offspring (vertical transmission). Furthermore, in some species, such as possibly the smooth-coated otter, older individuals may not be able to acquire new foraging skills as easily as younger otters, perhaps making them unsuitable for reintroduction programmes as their ability to adapt to their new environment and hence their survival chances may be limited. Finally, we found no direct evidence for the otters' reliance on social learning to depend on (assumed) foraging task complexity. However, future studies could present various types of appropriate live prey not normally provided in captivity (e.g. live fish, urchins, molluscs etc.) to assess social learning tendencies in such more ecologically relevant contexts.

In conclusion, this first exploration of social learning in otters shows that this taxon merits further study, not only because the wide range of life-history traits represented across the various species can provide further insights into the evolution of social learning strategies, but also because conservation efforts may be facilitated by an increased understanding of these species' ability to adapt to change through social transmission.

## Supplementary Material

Group compositions, solvers, discrete TADA results and video links

## Supplementary Material

Data

## Supplementary Material

NBDA code

## Supplementary Material

R input file

## Data accessibility

The data and R code are available as electronic supplementary materials, and data and electronic supplementary videos are available from the Dryad Digital Repository: http://dx.doi.org/10.5061/dryad.ct3s3 [41].
