# Supplementary material for: Social learning in otters
Source: R Soc Open Sci. 2017 Aug 30;4(8):170489. doi: 10.1098/rsos.170489 (PMC5579113; doi:10.1098/rsos.170489)
Supplement: Group compositions, solvers, discrete TADA results and video links [file rsos170489supp1.docx]

**SOCIAL LEARNING IN OTTERS**

**Zosia Ladds, William Hoppitt and Neeltje J. Boogert**

**SUPPLEMENTARY MATERIAL**

**Table S1.** Otter group compositions

| Group ID | Species | Location | Birthplace of Parents | No. of Males | No. of Females | Total otters |
| --- | --- | --- | --- | --- | --- | --- |
| SCG1 | Smooth-coated | Colchester Zoo | Cambodia  (Wild) | 3 (1 parent aged 8, 2 offspring aged 1) | 4 (1 parent aged 8, 3 offspring aged 2) | 7 |
| ASCG1 | Asian short-clawed | New Forest Wildlife Park | United Kingdom (Captivity) | 2 (1 parent aged 15, 1 offspring aged 9) | 3 (1 parent aged 14, 2 females aged 10) | 5 |
| ASCG2 | Asian short-clawed | New Forest Wildlife Park | United Kingdom (Captivity) | 4 (4 offspring aged 8-10) | 2 (2 offspring aged 8-10) | 6 |
| ASCG3 | Asian short-clawed | Paradise Wildlife Park | United Kingdom (Captivity) | 4 (1 parent aged 10, 3 offspring aged 4-6) | 2 (2 offspring aged 4-6) | 6 |

**Table S2.** Task presentation date & time, and

number of smooth-coated otters solving each task type

| Task Type | Presentation  Date & Time | No. of otters solved  (out of 7) |
| --- | --- | --- |
| 1 | 09/09/13, 10:30 AM | 7 |
| 2* | 11/09/13, 10:35 AM | 4 |
| 3 | 12/09/13, 10:20 AM | 5 |
| 4 | 17/09/13, 10:27 AM | 5 |
| 5 | 20/09/13, 10:10 AM | 7 |
| 6 | 25/09/13, 10:17 AM | 7 |

* Task 2 was presented over 2 experimental days. All other tasks were solved within 2 hours.

Tasks 5 and 6 were anticipated to be more difficult than tasks 1-4, so these data seem to contradict the theoretical prediction that social learning be used when asocial learning is costly or ineffective. However, here task difficulty was confounded with time, since tasks 5 and 6 were presented last, and this result was an ad hoc finding, not a test of an a priori hypothesis. Consequently, we designed the second experiment on Asian short-clawed otters such that task difficulty was balanced with order of presentation, so as to test for an analogous effect.

**Table S3.** Task presentation date & time, and number of Asian short-clawed otters

solving each task type

| Task Type | Presentation Date & Time  Group 1 | Presentation  Date & Time  Group 2 | Presentation  Date & Time  Group 3 | # of otters solved  (out of 17)  (Group 1/2/3) |
| --- | --- | --- | --- | --- |
| 1 | 10/07/2014  10:25 AM | 09/07/2014  12:20 AM * | 18/08/2014  12:20 PM | 13 (3/6/4) |
| 2 | 08/07/2014  10:40 AM | 07/07/2014  13:30 PM | 21/08/2014  12:17 PM | 13 (3/6/4) |
| 3 | 07/07/2014  10:37 AM | 08/07/2014  12:14 PM | 19/08/2014  13:15 PM * | 12 (3/6/3) |
| 4 | 09/07/2014  10:50 AM | 10/07/2014  12:15 PM | 20/08/2014  09:50 AM | 6 (4/0/2) |

* Task 1 for Group 2 and Task 3 for Group 3 were presented over 2 experimental days. All other tasks were solved within 4 hours.

The tasks were presented to the Asian short-clawed otter groups in a counter-balanced order across the three groups, with no one task occurring at the same position in the sequence, to avoid giving a prior advantage to solving one of the more difficult tasks.

**Inclusion of random effects for multiple diffusions**

If one runs an NBDA with multiple diffusions on the same individuals, a random effect of ‘individual’ should ideally be included [1]. However, this is currently only possible with the multiplicative OADA [2]. We wished to run our model averaging over both multiplicative and additive models to allow us to assess which model form was best supported, so this was not possible while including a random effect of individual. However, when we added an individual random effect to the multiplicative model it made very little difference (due to the diffusion taking a different path with each task). Thus, in this case, we could be confident in dropping the individual random effect from the analysis, and including both multiplicative and additive models in our model selection.

**Discrete time of acquisition diffusion analysis (dTADA)**

Order of acquisition diffusion analysis (OADA) is a variant of network-based diffusion analysis (NDBA) that analyses only the order in which individuals in a network acquire novel behaviour [2]. OADA has the advantage that it makes fewer assumptions than the alternative time of acquisition diffusion analysis (TADA) which takes into account the times at which novel behaviour is acquired. However, when networks are homogeneous, OADA has little power to distinguish social transmission from asocial learning, as seen in the wide confidence intervals obtained for Asian short-clawed otters (Main Text Table 2). To obtain more precise estimates of the strength of social transmission for this species, we ran a discrete time of acquisition diffusion analysis (dTADA; [2,3]. We could not run a continuous TADA because we were only able to record the minute in which each otter solved the task, not the exact times (see Main Text Methods). For the same reason we were unable to run a stratified OADA that takes into account the order of acquisition across diffusions [1].

The dTADA analysis proceeded in the same way as for OADA but with one additional aspect. A TADA is able to infer social transmission across multiple diffusions even if the network is homogeneous since social transmission would result in the novel behaviour diffusing at different times in each diffusion, depending on when innovation occurred [1]. However, to ensure that such an effect is not a spurious one, it is important to allow for the fact that different tasks might be of different difficulty [1], therefore we included “task” as a factor in the analysis.

The aim of the dTADA was to obtain more precise estimates of the effect of social transmission, and hence this is what we report in the Main Text. In Table S4 we give additional information on the results of the dTADA.

**Table S4.** A comparison of the support (based on Akaike weight) for the asocial learning model and different social learning models for the discrete TADA run on the task solves in the three groups of Asian short-clawed otters

| **Model form** | **Rate of social transmission same/different**  **across tasks** | **Support (total Akaike weight)** |
| --- | --- | --- |
| Asocial |  | 49.7% |
| Social transmission: |  |  |
| Additive model | Same | 24.8% |
|  | Different | 0.2% |
| Multiplicative model | Same | 24.8% |
|  | Different | 0.7% |

**VIDEOS**

**Video 1:** Smooth-coated otters are presented with task 5 (see main text Fig. 1)

Dryad Digital Repository. http://dx.doi.org/10.5061/dryad.ct3s3

**Video 2:** Asian short-clawed otters are presented with task 1 (see main text Fig. 2)

Dryad Digital Repository. http://dx.doi.org/10.5061/dryad.ct3s3

**Video 3:** Asian short-clawed otters are presented with task 4 (see main text Fig. 2)

Dryad Digital Repository. http://dx.doi.org/10.5061/dryad.ct3s3

**REFERENCES**

1. Hoppitt W, Laland KN. 2013 *Social Learning: An Introduction to Mechanisms, Methods and Models*. Princeton, USA: Princeton University Press.

2. Hoppitt W, Boogert NJ, Laland KN. 2010 Detecting social transmission in networks. *J. Theor. Biol.* **263**, 544–55. (doi:10.1016/j.jtbi.2010.01.004)

3. Franz M, Nunn CL. 2009 Network-based diffusion analysis: a new method for detecting social learning. *Proc. Biol. Sci.* **276**, 1829–36. (doi:10.1098/rspb.2008.1824)
